# Glycolytic lactate in diabetic kidney disease

**DOI:** 10.1172/jci.insight.168825

**Published:** 2024-06-10

**Authors:** Manjula Darshi, Luxcia Kugathasan, Soumya Maity, Vikas S. Sridhar, Roman Fernandez, Christine P. Limonte, Brian I. Grajeda, Afaf Saliba, Guanshi Zhang, Viktor R. Drel, Jiwan J. Kim, Richard Montellano, Jana Tumova, Daniel Montemayor, Zhu Wang, Jian-Jun Liu, Jiexun Wang, Bruce A. Perkins, Yuliya Lytvyn, Loki Natarajan, Su Chi Lim, Harold Feldman, Robert Toto, John R. Sedor, Jiten Patel, Sushrut S. Waikar, Julia Brown, Yahya Osman, Jiang He, Jing Chen, W. Brian Reeves, Ian H. de Boer, Sourav Roy, Volker Vallon, Stein Hallan, Jonathan A.L. Gelfond, David Z.I. Cherney, Kumar Sharma, for the Kidney Precision Medicine Project, and the CRIC Study Investigators

**Affiliations:** 1 Center for Precision Medicine, Division of Nephrology, Department of Medicine, University of Texas Health San Antonio, San Antonio, Texas, USA.; 2 Division of Nephrology, Department of Medicine, University Health Network, Toronto, Canada.; 3 Department of Population Health Sciences, University of Texas Health San Antonio, San Antonio, Texas, USA.; 4 Schools of Medicine and Public Health, University of Washington, Seattle, Washington, USA; 5 Department of Biological Sciences and Border Biomedical Research Center, University of Texas at El Paso, El Paso, Texas, USA.; 6 Clinical Research Unit, Khoo Teck Puat Hospital, Singapore.; 7 Herbert Wertheim School of Public Health, University of California, San Diego, La Jolla, California USA.; 8 Clinical Research Unit & Admiralty Medical Centre, Khoo Teck Puat Hospital, Singapore.; 9 Saw Swee Hock School of Public Heath, National University of Singapore, Singapore.; 10 Lee Kong Chian School of Medicine, Nanyang Technological University, Singapore.; 11 Center for Clinical Epidemiology and Biostatistics and; 12 Department of Biostatistics, Epidemiology, and Informatics, Perelman School of Medicine at the University of Pennsylvania, Philadelphia, Pennsylvania, USA.; 13 Department of Internal Medicine, University of Texas Southwestern Medical Center, Texas, USA.; 14 Glickman Urology and Kidney and Lerner Research Institutes, Cleveland Clinic, Cleveland, Ohio, USA.; 15 Section of Nephrology, Boston University School of Medicine and Boston Medical Center, Boston, Massachusetts, USA.; 16 Division of Nephrology, Department of Medicine, University of Illinois at Chicago, Chicago, Illinois, USA.; 17 Division of Nephrology, Department of Medicine, Wayne State University, Detroit, Michigan, USA.; 18 School of Public Health, Tulane University Medical Center, New Orleans, Louisiana, USA.; 19 Division of Nephrology, Department of Medicine, New Orleans, Louisiana, USA.; 20 Department of Medicine, University of California, San Diego, La Jolla, California, USA.; 21 VA San Diego Healthcare Center, San Diego, California, USA.; 22 Department of Clinical and Molecular Medicine, Faculty of Medicine, Norwegian University of Science and Technology, Trondheim, Norway.; 23 Department of Nephrology, St. Olav Hospital, Trondheim, Norway.

**Keywords:** Nephrology, Chronic kidney disease, Diabetes, Mitochondria

## Abstract

Lactate elevation is a well-characterized biomarker of mitochondrial dysfunction, but its role in diabetic kidney disease (DKD) is not well defined. Urine lactate was measured in patients with type 2 diabetes (T2D) in 3 cohorts (HUNT3, SMART2D, CRIC). Urine and plasma lactate were measured during euglycemic and hyperglycemic clamps in participants with type 1 diabetes (T1D). Patients in the HUNT3 cohort with DKD had elevated urine lactate levels compared with age- and sex-matched controls. In patients in the SMART2D and CRIC cohorts, the third tertile of urine lactate/creatinine was associated with more rapid estimated glomerular filtration rate decline, relative to first tertile. Patients with T1D demonstrated a strong association between glucose and lactate in both plasma and urine. Glucose-stimulated lactate likely derives in part from proximal tubular cells, since lactate production was attenuated with sodium-glucose cotransporter-2 (SGLT2) inhibition in kidney sections and in SGLT2-deficient mice. Several glycolytic genes were elevated in human diabetic proximal tubules. Lactate levels above 2.5 mM potently inhibited mitochondrial oxidative phosphorylation in human proximal tubule (HK2) cells. We conclude that increased lactate production under diabetic conditions can contribute to mitochondrial dysfunction and become a feed-forward component to DKD pathogenesis.

## Abstract 2

An elevated urine lactate-to-creatinine ratio suppresses mitochondrial function in diabetes and identifies individuals at risk of rapid renal decline independent of the level of chronic glycemic control.

## Introduction

The mechanisms responsible for diabetic kidney disease (DKD) remain incompletely understood and include metabolic abnormalities involving hyperlipidemia, sorbitol accumulation (1, 2), advanced glycation end product formation (3, 4), tubular growth (5), and glomerular hyperfiltration (5–8). These cellular and hemodynamic stressors may also drive inflammation and fibrosis (9). However, the underlying basis for cellular dysfunction from hyperglycemia remains unclear.

Recent systems biology-based analyses of multi-omics data, using metabolomics and transcriptomics data from patients with DKD, indicate that reduced mitochondrial function in cells unresponsive to insulin may be central to the cell dysfunction and consequent clinical complications (10, 11). Studies from experimental models of diabetes as well as patients with DKD have demonstrated a reduction of mitochondrial function (10, 12, 13). These prior experimental studies indicate that the onset of diabetes is associated with reduced mitochondrial content and electron transport activity in the diabetic kidney (12). Studies in patients with established DKD indicate a reduction of urinary metabolites associated with mitochondrial function and reduced levels of kidney peroxisome proliferator-activated receptor-γ coactivator (*PGC1A*), the master regulator of mitochondrial biogenesis (10). Reduced mitochondrial function is characteristically associated with a shift toward glycolysis resulting in elevated urine and plasma lactate levels and is used to diagnose patients with inborn errors of metabolism and severe mitochondrial dysfunction (14, 15). Lactate yielded from altered glycolysis in the defective mitochondria plays an important role in the progression of DKD; however, this relationship has only been partially established. In this study, we report on the role and regulation of lactate as a marker of kidney function decline in patients with diabetes. We also studied the role of the proximal tubule in lactate production and effects of lactate itself on mitochondrial function.

## Results

### Urine lactate is elevated in patients with type 2 diabetes and kidney disease as compared with healthy controls.

**Figure 1:**
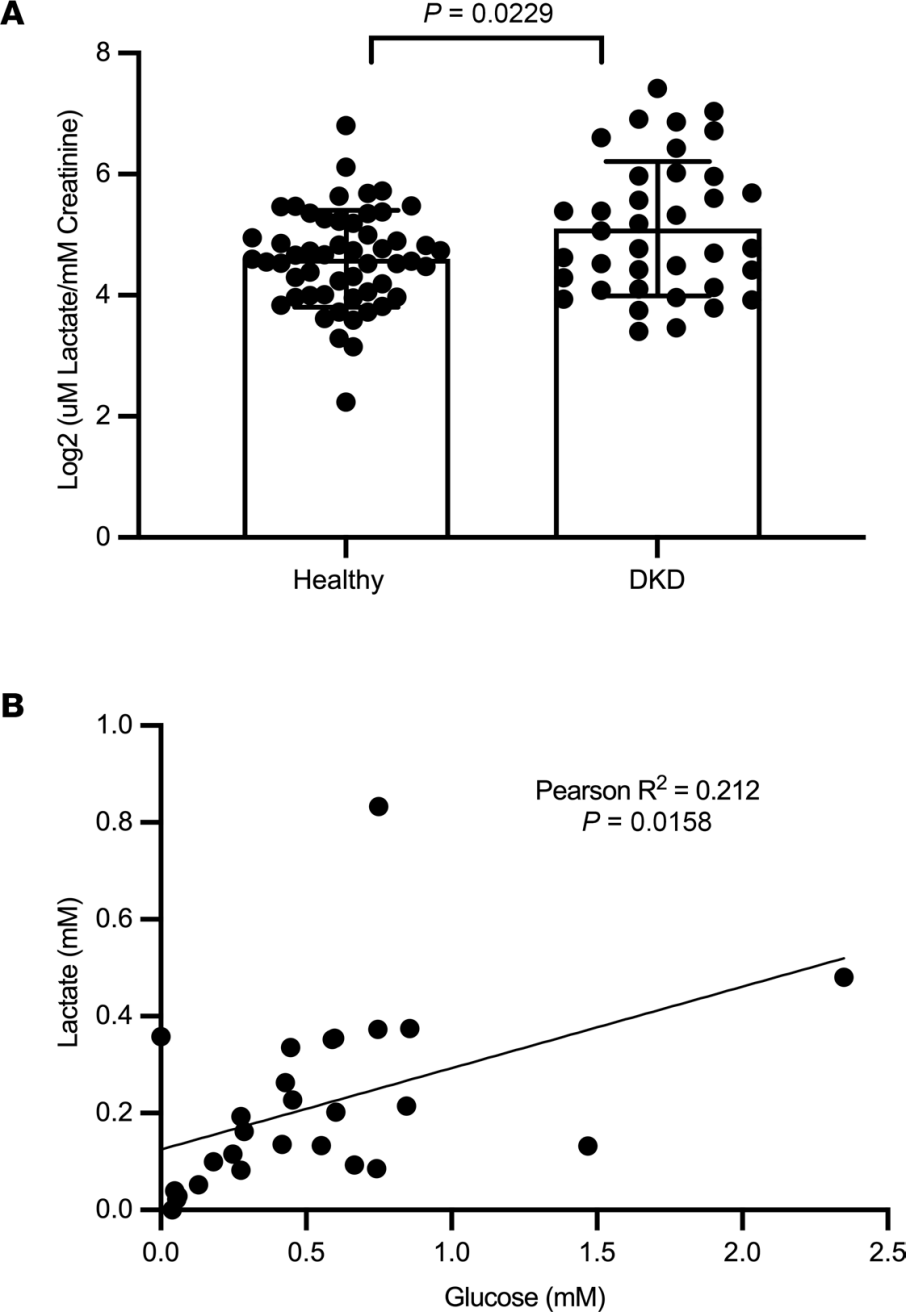
High urine lactate associated with diabetic kidney disease (DKD) among the HUNT3 cohort. (**A**) Lactate levels are significantly different between patients with DKD (eGFR < 60, *n* = 39) with age- and sex-matched healthy controls (*n* = 53) from the HUNT3 study, assessed using 2-tailed Welch’s *t* test. (**B**) Plasma lactate is correlated with plasma glucose in patients with DKD. Pearson’s correlation coefficient was used for correlation analysis. Data represent mean ± SD.

Urine samples from a subset of participants from the Nord-Trøndelag Health Study (HUNT3) study from Norway (16), in patients with type 2 diabetes (T2D) and established chronic kidney disease (CKD) (estimated glomerular filtration rate [eGFR] of < 60 mL/min/1.73 m^2^, *n* = 39) were analyzed for lactate and compared with age- and sex- matched healthy controls (*n* = 53). Baseline clinical characteristics of the study participants are shown in Table 1. Mean eGFR in healthy controls and DKD group was 88.0 ± 8.4 and 51.8 ± 8.3 mL/min/1.73m^2^, respectively. Urine lactate was elevated in patients with T2D and CKD (Figure 1A), and plasma lactate was positively associated with plasma glucose (Figure 1B) (*R*^2^ = 0.21, *P* = 0.016).

### High urine lactate is associated with faster decline in kidney function independent of the baseline level of eGFR.

To account for differences in urine lactate levels across ethnicities and regions, we obtained urine samples from 2 separate longitudinal studies, the Singapore Study of Macro-angiopathy and Micro-Vascular Reactivity in Type 2 Diabetes (SMART2D) study (17) based in Singapore and the Chronic Renal Insufficiency Cohort (CRIC) study (18, 19) based in the United States. Baseline characteristics of patients in SMART2D and CRIC cohorts are presented in Table 1. As shown in Table 2, eGFR slope in patients in the SMART2D cohort with urine lactate in the highest tertile (tertile 3) declined 1.63 mL/min/1.73m^2^ faster per year (95% CI, –2.85 to –0.46; *P* = 0.007) compared with those in the lowest tertile (tertile 1) after adjustment for baseline clinical risk factors including glycated hemoglobin A1c (HbA1c). The prognostic value of urine lactate levels was also assessed in baseline urine samples from 889 patients with T2D in the well-characterized CRIC study (20). Baseline characteristics of the CRIC study cohort are shown in Table 1. The eGFR slope in patients with urine lactate levels in tertile 3 declined 0.63 mL/min/1.73m^2^ (95% CI, –1.2 to –0.02; *P* = 0.04) faster per year compared with those in tertile 1 after adjustment for baseline clinical risk factors (Table 2).

### Hyperglycemia stimulates plasma and urine lactate levels.

**Figure 2:**
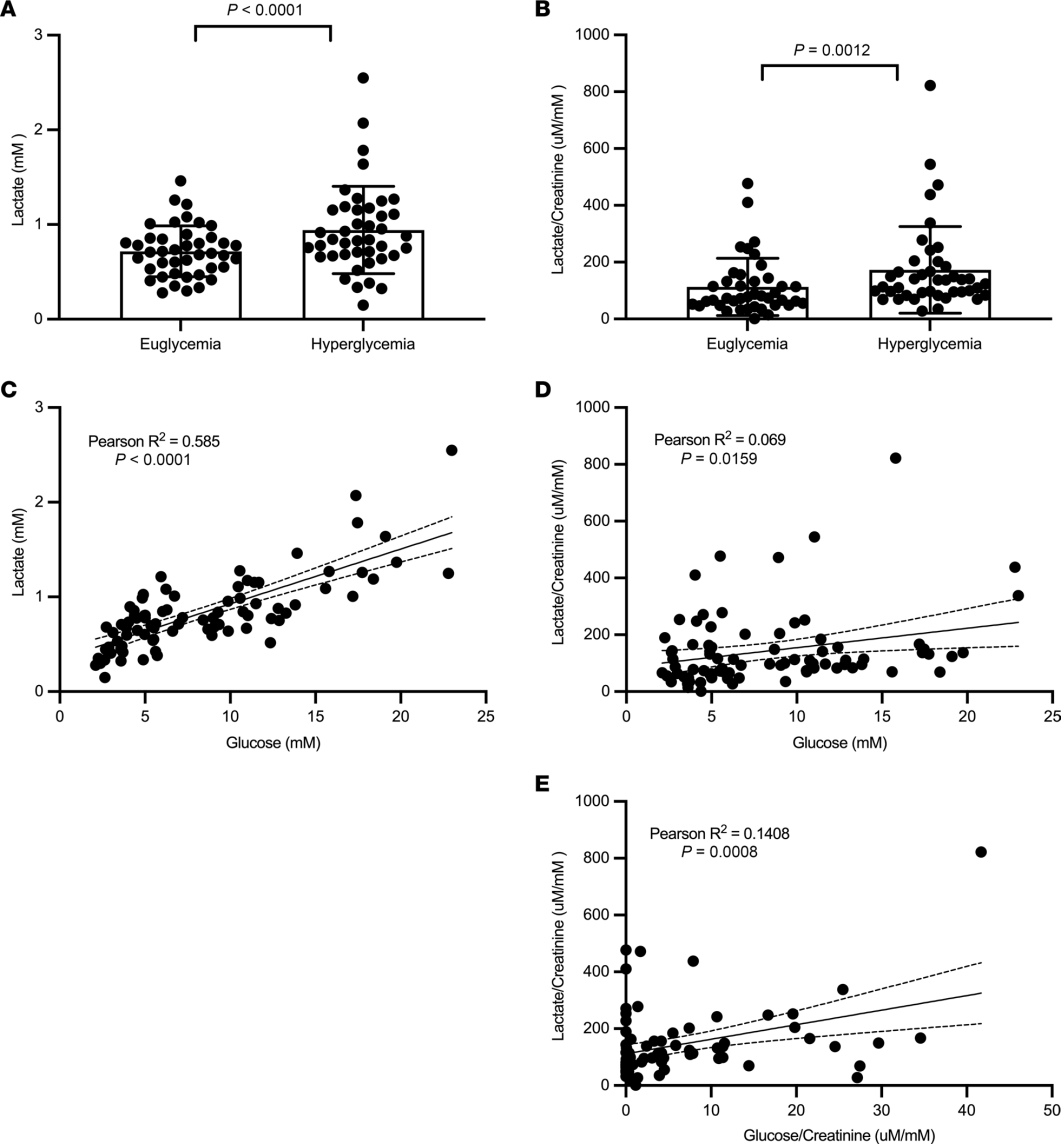
Urine lactate and plasma lactate increased with hyperglycemic clamp in T1D. Patients with T1D (*n* = 40) and with no evidence of kidney disease were studied with a glycemic clamp to maintain blood glucose levels at a 4–6 mM range or in a hyperglycemic range (9–11 mM). (**A** and **B**) The changes in plasma and urine lactate/creatinine are demonstrated following the euglycemic and hyperglycemic clamps. *P* values in panels **A** and **B** were calculated by Wilcoxon matched-pairs signed rank test. (**C** and **D**) Both plasma and urine lactate are correlated with plasma glucose. (**E**) Urine lactate is correlated with urine glucose. Correlation analyses were performed using the Pearson’s correlation coefficient. Data represent mean ± SD.

To determine whether hyperglycemia was a major factor in regulating lactate levels, we analyzed lactate levels in blood and urine in a type 1 diabetes (T1D) cohort from the ATIRMA study who underwent glycemic clamp studies (21). Blood glucose levels were maintained between 4 and 6 mM by euglycemic clamp and later between 9 and 11 mM by hyperglycemic clamp (22). There was a robust intraindividual increase in plasma and urine lactate with the hyperglycemic clamp (Figure 2, A and B). Additionally, there was a strong correlation (Pearson *R*^2^ of 0.585, *P* < 0.0001) with plasma glucose and plasma lactate levels (Figure 2C). Among patients with plasma glucose above 15 mM, the plasma lactate levels ranged between 0.98 and 2.55 mM. Urine lactate levels were modestly yet significantly correlated with plasma glucose levels (Pearson *R*^2^ of 0.069, *P* = 0.0159) (Figure 2D), while the strength of correlation of urine lactate was greater with urine glucose levels (Pearson *R*^2^ of 0.1408, *P* = 0.0008) (Figure 2E).

### Elevated lactate is contributed by renal cells.

**Figure 3:**
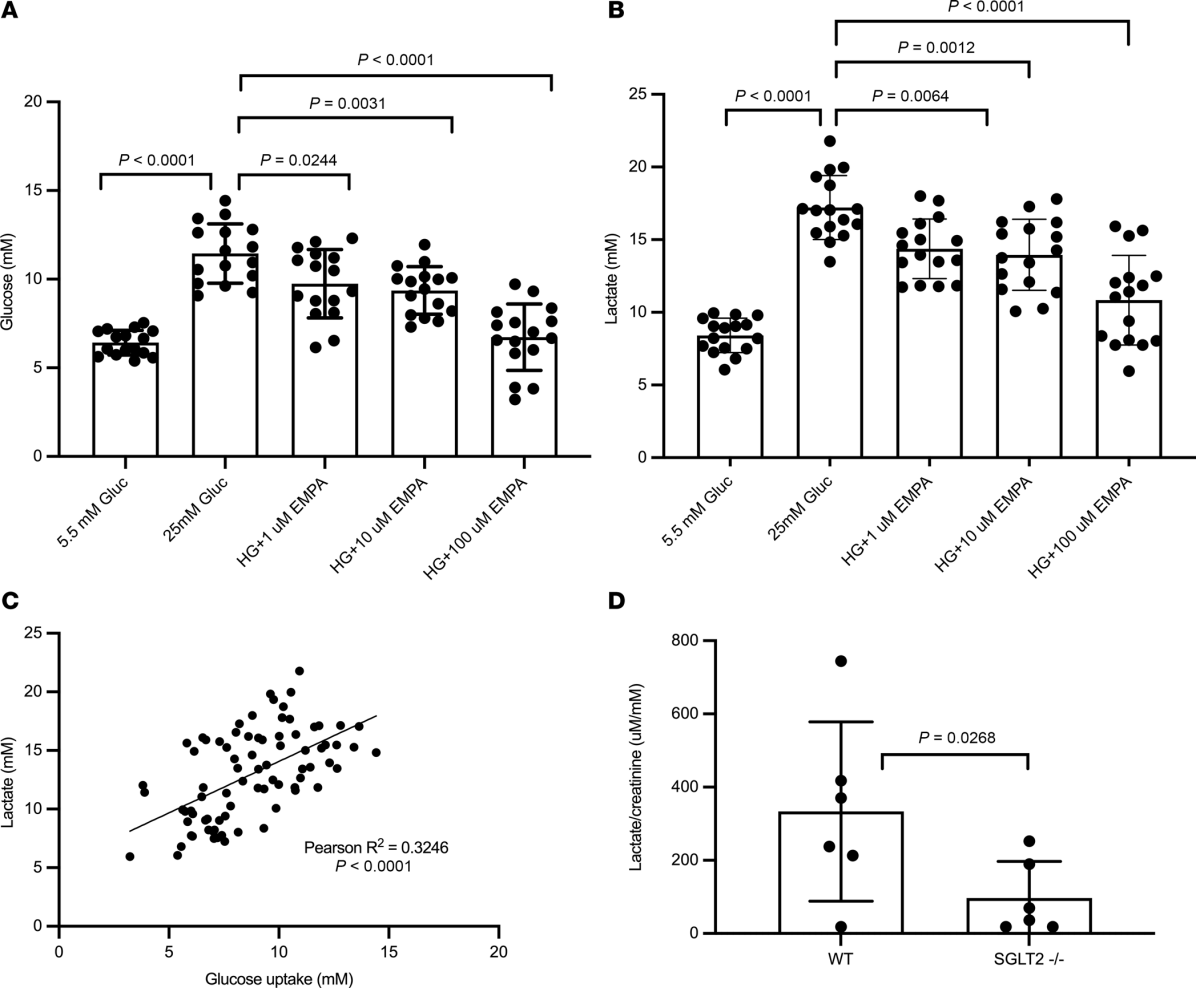
Glucose uptake and lactate secretion in isolated kidney sections. (**A** and **B**) Kidney sections (*n* = 16) from 10- to 12-week-old male C57BL/6J mice have an SGLT2-dependent increase in glucose uptake (**A**) from normal (NG) to high glucose (HG) and lactate production (**B**). The SGLT2 inhibitor empagliflozin (EMPA) has a dose-dependent effect to reduce glucose uptake and lactate secretion. *P* values for **A** and **B** were calculated using 1-way ANOVA and Tukey’s test for multiple comparison testing. (**C**) Lactate secretion is significantly correlated with glucose uptake, assessed using the Pearson’s correlation coefficient. (**D**) Urine lactate is reduced in SGLT2-KO (SGLT2^–/–^) mice via *t* test (7-month-old male mice, *n* = 6 both groups) compared with WT by 2-tailed Welch’s *t* test. Data represent mean ± SD.

To determine if glucose exposure within renal parenchyma contributes to lactate production in the kidney, we studied ex vivo mouse kidney tissue sections exposed to normal glucose (NG) or high glucose (HG) for up to 24 hours. Previous studies have demonstrated that mouse kidney tissue sections are viable for up to 72 hours and can be used as a valuable ex vivo model for renal fibrosis (23). Tissue sections of 100 μm thickness were incubated for 24 hours in media containing 7.2 mM glucose (NG) or 25 mM glucose (HG). There was a significant increase in glucose uptake (*P* < 0.0001; Figure 3A) and a stimulation of lactate secretion (*P* < 0.0001; Figure 3B) following 24-hour incubation in HG relative to NG media. Lactate levels were closely correlated with glucose uptake (Pearson *R*^2^ of 0.325, *P* < 0.0001), suggesting that kidney cells can generate high amounts of tissue lactate in response to HG exposure (Figure 3C). The elevated lactate levels are likely arising in part from proximal tubular cells, since exposure of kidney slices to sodium-glucose cotransporter-2 (SGLT2) inhibitors (empagliflozin, dapagliflozin, and canagliflozin) attenuated glucose uptake and reduced lactate secretion (Figure 3, A and B, and Supplemental Figure 1; supplemental material available online with this article; https://doi.org/10.1172/jci.insight.168825DS1). Furthermore, urine levels of lactate were reduced in SGLT2-KO mice as compared with age- and sex- matched WT controls (Figure 3D).

### Elevation of glycolytic genes in human diabetic kidneys.

**Figure 4:**
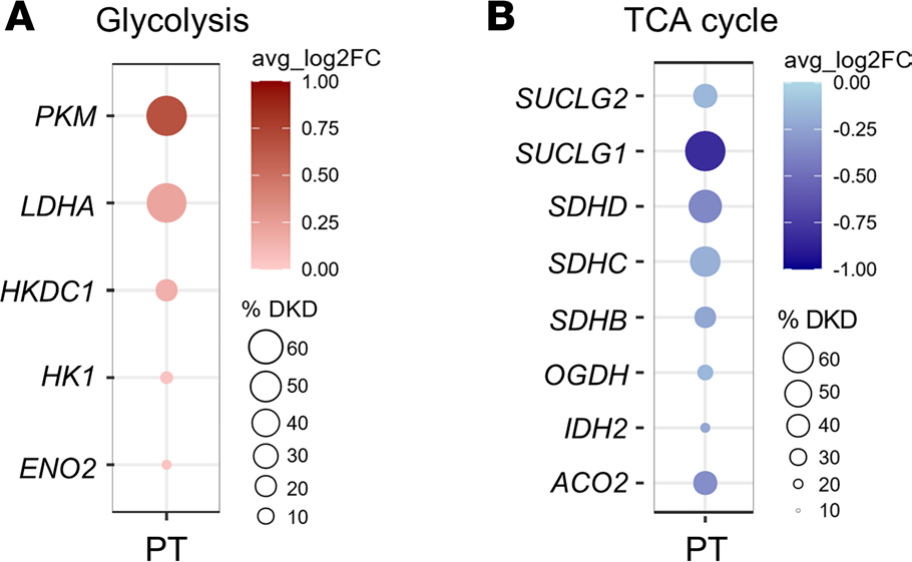
Dot plot of upregulated glycolytic and downregulated TCA cycle genes in the proximal tubular (PT) cells of patients with diabetic kidney disease (DKD). (**A** and **B**) Log_2_ fold change calculated between the average of normalized glycolytic (**A**) and TCA cycle (**B**) gene expression values from the living donors (LD; *n* = 20) and patients with DKD (*n* = 11) in PT cells. The %DKD circle size shows the percentage of cells in which the gene was detected in DKD biopsies. PT, proximal tubule; *PKM*, pyruvate kinase; *LDHA*, lactate dehydrogenase A; *HKDC1*, hexokinase domain containing 1; *HK1*, hexokinase 1; *ENO2*, enolase 2; *SUCLG2*, succinate-CoA ligase GDP-forming subunit β; *SUCLG1*, succinate-CoA ligase GDP/ADP-forming subunit α; *SDHD*, succinate dehydrogenase complex subunit D; *SDHC*, succinate dehydrogenase complex subunit C; *SDHB*, succinate dehydrogenase complex subunit B; *OGDH*, oxoglutarate dehydrogenase; *IDH2,* isocitrate dehydrogenase (NADP^+^)2; *ACO2*, aconitase 2.

To determine if glycolysis, a source of lactate production, is enhanced in the human diabetic kidney, we compared kidney tissue cell-specific glycolytic gene expression using single-cell RNA-Seq (scRNA-Seq) between adults with diabetes (*n* = 11) and healthy controls (*n* = 20) from the Kidney Precision Medicine Project (KPMP) Kidney Tissue Atlas (Supplemental Table 1). Several genes involved in early- and late-stage glycolysis were significantly upregulated in the proximal tubule of participants with DKD, including pyruvate kinase (*PKM*), lactate dehydrogenase A (*LDHA*), and hexokinase domain containing 1 (*HKDC1*), compared with healthy controls (Figure 4A and Table 3). Moreover, the majority of the TCA cycle (*n* = 8) and respiratory genes (*n* = 35) were significantly downregulated in the proximal tubule of patients with DKD compared with healthy individuals (Figure 4B and Supplemental Figure 2), suggesting that elevated glycolysis and suppressed TCA cycle–mediated energy production may contribute to lactate overproduction in the proximal tubular cells in diabetes.

### Lactate inhibits mitochondrial oxygen consumption rates in kidney proximal tubule epithelial cells.

**Figure 5:**
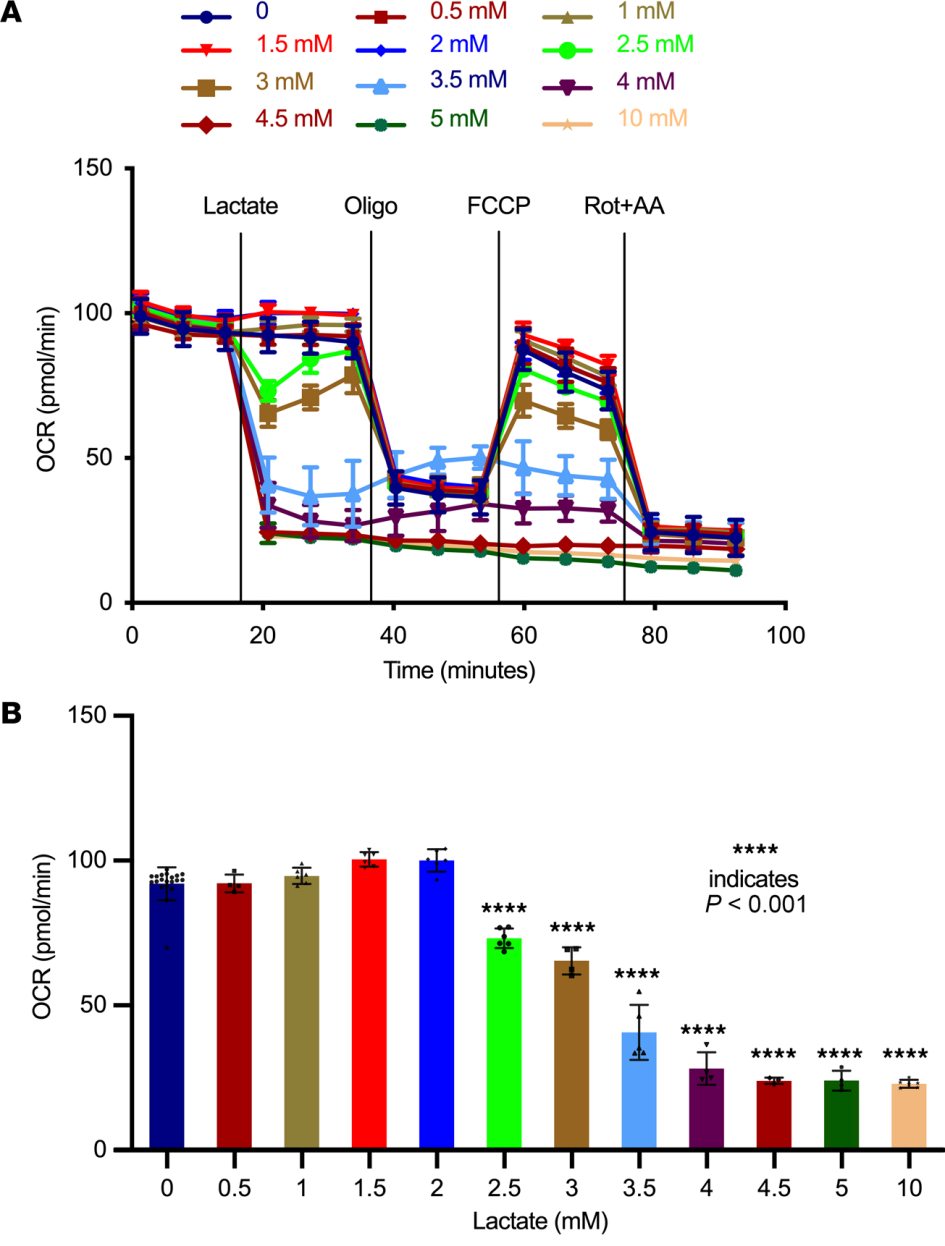
Lactate inhibits mitochondrial function in a dose-dependent manner in human kidney proximal tubule epithelial cells (HK2). (**A**) Oxygen consumption rates (OCR) were measured in HK2 cells (*n* = 15) using seahorse extracellular flux analyzer. Following basal respiratory measurements, indicated concentrations of lactic acid were injected through injection ports. (**B**) Basal respiration is represented immediately after treatment with lactate (*n* = 20). *P* values in **A** were calculated using 1-way ANOVA and Dunnett’s test for multiple comparison testing. Data represent mean ± SD. Oligo, oligomycin; FCCP, carbonyl cyanide-p-trifluoromethoxyphenylhydrazone; Rot+AA, Rotenone+Antimycin A.

To understand the effect of increased lactate, we measured mitochondrial function in human kidney epithelial (HK2) cells acutely treated with increasing concentrations of lactate (Figure 5, A and B). With slightly elevated levels of lactate between 0.5 and 2.0 mM L-lactate, there was an increase in oxygen consumption rate (OCR), indicating an increase in mitochondrial respiration. However, with lactate levels ≥ 2.5 mM, there was a marked reduction of both basal and maximal OCRs in a dose-dependent manner within 6 minutes of exposure. In addition, 1 hour of lactate preincubation in isolated mouse primary renal proximal tubular epithelial cells (pTECs) showed dose-dependent inhibition of OCR. Basal respiration, maximal respiration, and ATP-linked OCR were significantly decreased from 2 mM extracellular lactate exposure (Supplemental Figure 3A). Moreover, MTT assay in HK2 cells exposed to various concentrations of lactate demonstrated that lactate has no cytotoxicity up to 5 mM (Supplemental Figure 3B)

### Intracellular lactate accumulation supresses ATP production in diabetic kidneys.

**Figure 6:**
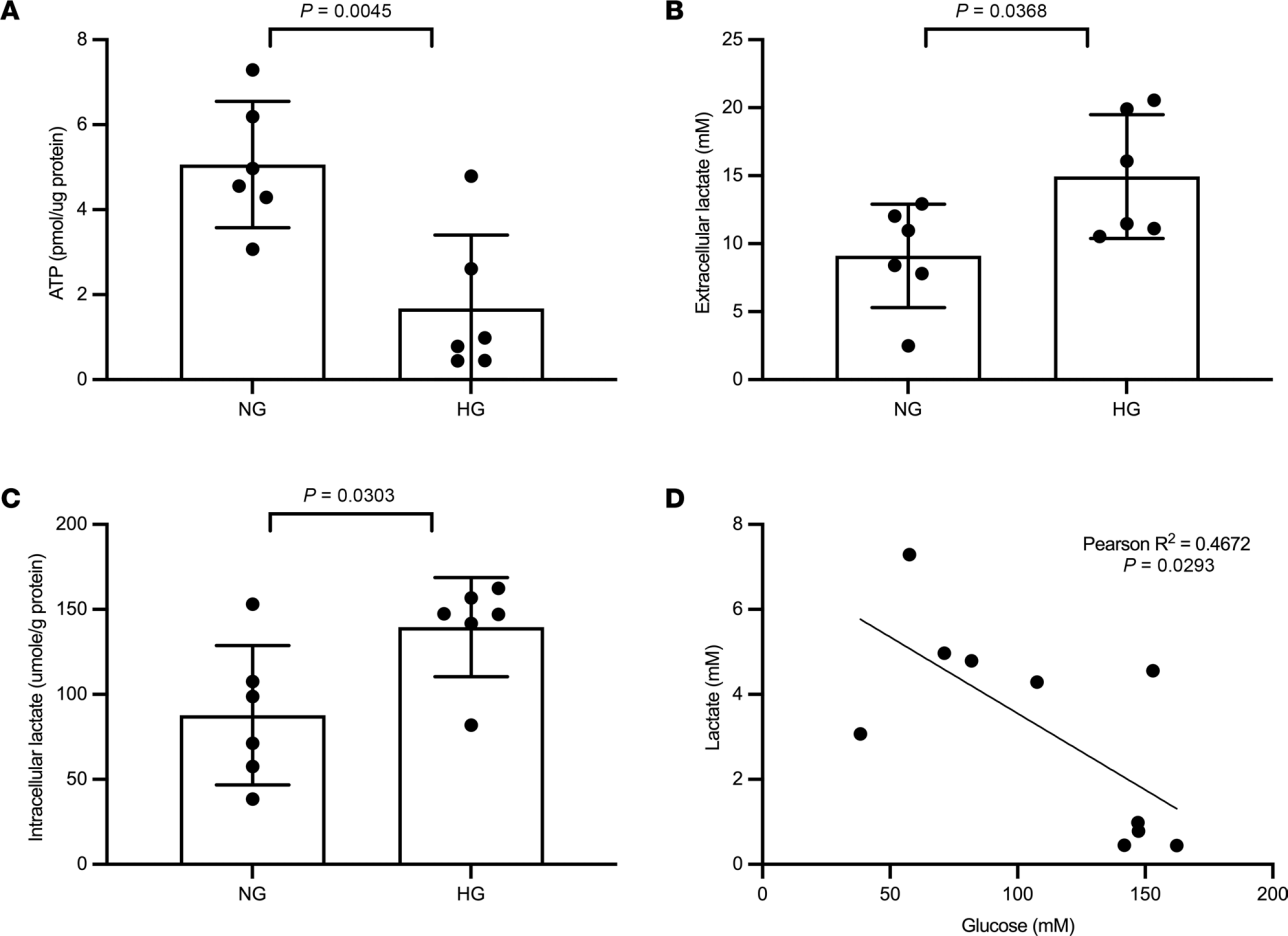
ATP production and intracellular lactate accumulation in isolated kidney sections. (**A**–**C**) Kidney sections (*n* = 5–6 each group) from 10- to 12-week-old male C57BL/6J mice were incubated with normal glucose (NG) and high glucose (HG) for 24 hours to measure the ATP level (**A**), extracellular lactate (**B**), and intracellular lactate (**C**). (**D**) The ATP level negatively correlates with intracellular lactate (*n* = 10). Two-tailed *t* test in **A**–**C** and simple linear regression analysis in **D** using the Pearson’s correlation coefficient were performed for the statistical analyses. Data represent mean ± SD.

To investigate whether intracellular lactate accumulation affects ATP production in tubular cells, mouse kidney cortical sections (100 μm) were incubated with normal and HG for 24 hours. ATP (Figure 6A), extracellular lactate excretion (Figure 6B), and intracellular lactate (Figure 6C) levels were measured in the lysate of the kidney sections. In addition to the increased extracellular lactate, ATP production was significantly decreased with elevation of intracellular lactate upon HG exposure. Importantly, the intracellular lactate accumulation showed a significant inverse correlation with ATP (Figure 6D). Since plasma membrane lactate transporters (MCT) mediate the intracellular lactate clearance, we checked the expression of MCTs in the proximal tubule of patients with diabetes. Among them, the bidirectional plasma MCT, MCT1 (24, 25), was significantly downregulated, whereas MCT2 was upregulated (Supplemental Figure 4A). To evaluate the role of MCT1 in intracellular lactate accumulation, kidney cortical sections were treated with a small molecule MCT1 inhibitor. The elevated intracellular lactate upon MCT1 inhibition (Supplemental Figure 4B) suggests that impaired lactate clearance may also contribute to lactate accumulation in the diabetic kidney. Moreover, while nondiabetic db/m mice demonstrate similar concentrations of lactate levels at the kidney cortex and medulla, diabetic db/db mice show significantly higher levels at the cortex (Supplemental Figure 5), indicating a large degree of heterogeneity with lactate production in different regions and cell types within the kidney. Altogether, altered lactate production in kidney resident cells with intracellular lactate accumulation is associated with reduced mitochondrial bioenergetics in the diabetic kidney.

## Discussion

The basis for progressive reduction of organ function with chronic diabetes may reside in progressive mitochondrial dysfunction and failure. The present study indicates that higher urine lactate/creatinine ratio is associated with greater eGFR decline in patients with T2D and CKD. Furthermore, we demonstrate a strong correlation between glucose exposure and plasma and urine lactate levels in adults with T1D undergoing a hyperglycemic clamp. Interestingly, we observed that lactate is released from mouse kidney sections, regulated by SGLT2 inhibitors, and that glycolytic genes are upregulated in the proximal tubule of the human diabetic kidney. Lastly, we showed that lactate itself could directly contribute to suppressed mitochondrial bioenergetics in proximal tubular cells and in diabetic kidney sections.

In the cross-sectional study of a Norwegian community population, HUNT3 patients with T2D and CKD (eGFR < 60 mL/min/1.73m^2^) had higher levels of urine lactate compared with matched healthy controls. In the Asian longitudinal SMART2D cohort (composed of 3 different ethnicities) and in the CRIC United States–based cohort, patients in the third tertile of urine lactate had faster rates of eGFR decline than those in the first tertile. To assess whether urine lactate at lower eGFR levels is a marker of renal injury as opposed to just a marker of lower kidney function, we restricted analysis to participants with eGFR greater than 45 mL/min/1.73m^2^ in the SMART2D cohort. Despite restriction, the remaining subset of participants still displayed a significantly faster rate of eGFR decline in the third tertile of urine lactate than those in the first tertile. Consistent with our data, a recent cross-sectional study observed elevated urinary lactate levels in patients with DKD (26). Thus, our data from cross sectional and longitudinal cohort studies support the association of urine lactate level with kidney dysfunction and its subsequent decline.

Given that urine lactate generation could be controlled by a variety of factors, we examined whether elevations in blood glucose could be a causative factor. In the current analyses, euglycemic and hyperglycemic clamp studies demonstrated that increasing blood glucose from 3–6 mM to 9–11 mM increased lactate levels in blood and urine. Additionally, urine lactate was moderately correlated with urine glucose (*R*^2^ = 0.1408, *P* = 0.0008) indicating that the local microenvironment of glucose levels in the urine space — and, by extension, kidney tubules — is likely an important stimulus for urine lactate level.

To determine if the kidney contributes to lactate production, we found a close correlation between glucose uptake in kidney tissue and lactate levels in the supernatant, demonstrating that glucose uptake is sufficient to enhance lactate production in kidney cortical cells. Since SGLT2 inhibition reduced lactate levels, there is a potential role for glucose uptake via SGLT2 in proximal tubular cells in regulating lactate production. Moreover, SGLT2-KO mice exhibited lower urine lactate levels than control mice, suggesting that glucose uptake via the SGLT2 transporter contributes to urine lactate levels under physiologic conditions. A recent study investigating the effect of SGLT2 inhibition on scRNA-Seq data in kidney biopsies of patients with youth-onset T2D found suppression of glycolytic gene expression with treatment, including aldolase B and phosphofructokinase (27). Although the degree of glycolytic activity in proximal tubular cells is still disputed (28, 29), using kidney tissue scRNA-Seq data from the KPMP Kidney Tissue Atlas (30), we observed higher expression of several glycolytic genes, including *PKM*, *LDHA*, and *HKDC1* in the proximal tubule from participants with diabetes compared with healthy controls. In contrast, most of the TCA cycle and respiratory genes are downregulated in the proximal tubule of patients with diabetes compared with healthy controls. Of note, prior studies using proteomics identified PKM2 to be increased in the diabetic kidney of patients with T1D (31, 32). Indeed, *PKM* was the most significantly upregulated gene in the proximal tubule of the diabetic kidney. We also found an increase in *HKDC1*, which is a recently discovered hexokinase increased in various cancer cells. A possible source of enhanced lactate production from the human kidney is *LDHA*, which was significantly increased in the proximal tubular cell of the human diabetic kidney. As well as under diabetic conditions, this apparent glycolytic shift is also demonstrated following ischemic acute kidney injury, as seen by an acute increase in kidney lactate levels and hexokinase activity (33). Elevated *LDHA* levels have also been associated with patients with DKD (26). Given our understanding of the relationship between glucose uptake and lactate production, we speculate that there is a glycolytic shift in proximal tubular cells in patients with DKD. The downregulation of the TCA cycle might trigger the glycolytic shift to compensate for the mitochondrial respiration–derived energy deficiency in the diabetic kidney (34). Whether the switch to glycolysis in proximal tubular cells is beneficial or harmful in patients with longstanding diabetes remains to be determined in dedicated studies. Of note, a prior study found that a switch to glycolysis could be protective, especially in podocytes (31, 32).

Under suppression of TCA cycle and respiration, the production of lactate may indicate that renal tubular cells in the diabetic milieu have shifted from mitochondrial oxidative phosphorylation to a predominantly glycolytic metabolism. Data to support this conclusion have also arisen from several studies in mouse models of T1D and T2D (12, 13, 35). Specifically, reductions in oxidative phosphorylation activity in experimental DKD models have been demonstrated at the protein level and at the functional level (13). In vivo metabolic flux studies have also demonstrated an increase in kidney lactate derived from labeled glucose in T2D mice (13). In addition, urine metabolomics indicated an overall reduction in mitochondrial function in patients with DKD with both T1D and T2D (10). The cumulative results of these studies indicate that the diabetic kidney undergoes an increase in glycolysis and that urine lactate may be a marker of glycolysis activity and reduced mitochondrial oxidative phosphorylation activity (36).

Exposure of proximal tubular cells to exogenous lactate is sufficient to reduce oxidative phosphorylation activity and may contribute to the Warburg-like effect observed in diabetic kidney tissue (13, 37). Though the exact mechanism of this preferential switch to glycolysis is incompletely understood, it is compatible with mitochondrial dysfunction. The glycolytic intermediates generated may be shunted to alternative pathways (e.g., pentophosphate), known to be responsible for diabetic vascular injury (38). To our knowledge, this is the first demonstration that exogenous lactate inhibits mitochondrial function in kidney cells. Whether lactate above the physiological range (≥2 mM) affects mitochondrial dysfunction in the setting of DKD remains to be established. However, the mechanism behind lactate-induced mitochondrial bioenergetic suppression was recently demonstrated. Daw et al. showed that lactate induces rapid Mg^2+^ release from the endoplasmic reticulum and subsequent mitochondrial uptake (39). In hepatocytes, 5 mM extracellular lactate inhibited mitochondrial oxidative phosphorylation due to elevated mitochondrial Mg^2+^ levels, and inhibition of mitochondrial Mg^2+^ uptake may be organ protective (39). The requirement of exogenous lactate to suppress mitochondrial bioenergetics varies with cell type due to their differential lactate influx. Based on our data, 2–2.5 mM is sufficient to reduce oxidative phosphorylation in mouse and human proximal tubular cells. Local lactate levels at the tissue microenvironment may be much greater than circulating levels (36). The observed negative correlation between HG-induced intracellular lactate accumulation and ATP level in kidney cortical sections supports the hypothesis that lactate contributes to mitochondrial dysfunction in the diabetic kidney. A prior study reported that fasting plasma lactate concentration in patients with T2D is 1.46 ± 0.14 mM and 0.81 ± 0.07 mM in healthy controls (40). The level of 1.46 mM in patients with T2D is approaching the level of lactate our data presents in order to induce impairments in mitochondrial respiration for kidney cells. Indeed, in tumor tissue, lactate levels could exceed 40 mM (41) and portend a poor prognosis for overall survival. A prior study indicated that lactate levels above 10 mM are immune suppressive to cytotoxic T cells (36). Similar to the tumor microenvironment, the diabetic kidney is also characterized by hypoxia (42). This is consistent with our interpretation that increased levels of urine lactate indicate a shift from oxidative phosphorylation to glycolysis. Taken together, the experiments conducted in this study confirm that mitochondrial function in kidney proximal tubular cells are sensitive to elevated lactate levels and that urine lactate levels could be used as an indicator of excess lactate production in the kidney with prognostic implications for subsequent kidney function decline.

Strengths of our study include the (a) evaluation of urine lactate in 4 distinct cohorts of patients with diabetes, (b) hyperglycemic clamp studies indicating that urine lactate production is secondary to hyperglycemia, (c) orthogonal data in mouse samples demonstrating that the kidney proximal tubular cell is a potential source of lactate production in response to excess glucose, and (d) demonstrating that lactate accumulation suppresses mitochondrial function in kidney proximal tubular cells. The role of urine lactate for other systemic complications of diabetes remains to be established. Further studies are required to determine whether urine lactate levels reflect kidney mitochondrial dysfunction directly and whether this same mechanism is causally associated with other organ dysfunctions in diabetes such as retinopathy or neuropathy.

In conclusion, our translational experiments in well-characterized patients with T2D indicate that urine lactate is a marker for progressive decline in kidney function. We further demonstrate that renal lactate is stimulated by glucose and may contribute to reduced mitochondrial function as a contributor to DKD and loss of kidney function.

## Methods

### Sex as a biological variable.

Our HUNT3, SMART2D, and CRIC trial cohorts examined male and female participants, but sex was not considered a biological variable in our study. Our animal studies exclusively examined male mice because male animals exhibited less variability in phenotype. It is unknown whether the findings are relevant for female mice.

### Study populations and study design.

We took advantage of characteristics of 4 study populations; this included a cross-sectional analysis in a T2D cohort to determine association of lactate with current eGFR, 2 longitudinal cohorts in T2D to determine association with change in eGFR, and finally a mechanistic study in T1D that examined exposure to 2 levels of clamped glycemia.

We measured lactate in individuals with T2D from fasting spot urine samples or timed collections in 3 independent cohorts: HUNT3 from Norway (16), SMART2D from Singapore (17), and CRIC from the United States (18, 19). Participants from the community-based HUNT3 study included 37 individuals with T2D and reduced eGFR (eGFR < 60 mL/min/1.73m^2^) and 53 age- and sex- matched healthy controls without diabetes or reduced eGFR. SMART2D involved participants with T2D who underwent a hospital-based research visit every 3 years and whose kidney function was regularly captured using electronic medical records. Urine lactate was measured in 576 participants in the SMART2D cohort in an earlier study (43). Those with baseline eGFR between 30 and 90 mL/min/1.73m^2^ (*n* = 230, CKD stage 2 and 3) were included in the current analysis. The CRIC study design and methods have been published earlier (18–20). Briefly, CRIC is a prospective longitudinal cohort study of patients with existing kidney disease who have been followed yearly for up to 10 years. The participants had established CKD with eGFR < 75 mL/min/1.73 m^2^. We included 889 participants with T2D who had baseline 24-hour urine collections.

Urine and plasma lactate were also measured under controlled euglycemic and hyperglycemic clamp conditions from a previously published clinical study in patients with T1D (*n* = 42) with or without renal hyperfiltration in the ATIRMA study (21). Euglycemic clamp (4–6 mM glucose) conditions were maintained for approximately 4 hours prior to urine or blood collection. The following day, hyperglycemia (9–11 mM glucose) was maintained for 4 hours. Urine and blood samples for lactate measurements were performed from samples obtained at the 4-hour time point following euglycemia or hyperglycemia.

### Analyte measurements.

Plasma and urine glucose and lactate were measured with the YSI 2700 biochemistry analyzer in a 96-well format. Urine creatinine was measured by creatinine assay kit from Cayman Chemicals. Urine lactate levels in the SMART2D cohort was measured by gas chromatography–mass spectrometry as described previously (43).

### Mouse studies.

Mouse kidney sections were prepared as described previously, with minor modifications (23). Briefly, male C57BL/6J mice at 10–12 weeks of age were euthanized by cervical dislocation, and kidneys were decapsulated, washed, and sectioned in PBS into 100 μm slices using OTS-500 Vibratome (Electron microscopy science). PBS-washed sections were transferred into 24-well plate and incubated at 37°C and 5% CO_2_ with 500 μL of renal epithelial cell growth media (ATCC, PCS-400-040) with NG (7.2 mM) or HG (25 mM) or MCT inhibitor (MilliporeSigma, SR13800) as indicated. Conditioned media were collected after 24 hours for measurements of glucose uptake and lactate secretion in the presence and absence of SGLT2 inhibitors (dapagliflozin and canagliflozin). Spot urine was collected in SGLT2-KO and littermate WT mice (*n* = 6/group, all male, 7 months of age). *Sglt2* (*Slc5a2*) mutant mice were generated at Lexicon Pharmaceuticals and has been previously described (44). Urine lactate was normalized to urine creatinine concentration. No significant difference in body weight and blood glucose was observed between C57BL/6 SGLT2-KO and C57BL/6 WT mice (28.4 ± 0.6 g versus 29.6 ± 0.6 g and 121 ± 6 mg/dL versus 124 ± 5 mg/dL, respectively; both NS).

### ATP and intracellular lactate measurement.

ATP was measured in mouse kidney sections using a fluorometric assay kit (MilliporeSigma, MAK190). In brief, kidney sections were incubated in 500 μL of renal epithelial cell growth media with NG (7.2 mM) or HG (25 mM) for 24 hours. After washing twice with PBS, sections underwent lysis, and ATP was measured in the lysate according to the manufactured protocol. Protein in the lysate was estimated using BCA Protein Assay kit (Thermo Fisher Scientific, 23227) to express the ATP level as pmol/μg of protein. Intracellular lactate was measured in the lysate of the kidney sections incubated with or without MCT inhibitor or kidney cortex and medulla according to the manufacture’s protocol (L-lactate assay kit; Cayman Chemical, 700510). This study used kidney sections from 10- to 12-week-old male C57BL/6J mice and the kidney cortex and medulla from 6 months C57BLKS/J nondiabetic db/m and diabetic db/db mice (*n* = 5/group).

### Isolation of mouse pTECs.

pTECs were isolated using 8- to 10-week-old C57BL/6 male mice using established protocols (45) with slight modifications described in detail in a previous study (46). Kidney cortex was isolated, chopped, and washed with PBS including 10% penicillin-streptomycin (Thermo Fisher Scientific, 15140122). The tissues were digested in collagenase IV (MilliporeSigma) for 30 minutes at 37°C. The digested cortical tissue was then filtered via 70 μm filter and resuspended in renal epithelial growth medium (REGM) and centrifuged at 50*g* for 5 minutes at room temperature. The pellet was resuspended in REGM and plated onto a collagen-coated T25 flask. All experiments were performed at passage 1.

### Mitochondrial function measurements in HK2 and isolated mouse primary renal pTECs.

HK2 cells (ATCC, CRL-2190) were maintained in keratinocyte serum free media (Thermo Fisher Scientific) at 37°C and 5% CO_2_ conditions. No mycoplasma contamination was detected during quality control of the HK2 cells. For mitochondrial functional measurements, 40,000 cells/well were plated on Seahorse XF-96 plates 48 hours prior to the assay in culture media. One hour prior to the assay, cells were washed and incubated with assay media (XF media, pH 7.4, supplemented with 5 mM HEPES) for 1 hour in a CO_2_ free incubator. Following basal respiration, lactate diluted in assay media or media control was injected through the XF-cartridge injection port and respiratory rates were collected as above. Isolated mouse pTECs were incubated with various concentration of lactate for 1 hour in a CO_2_ free incubator before the Seahorse assay. To measure maximal respiration and mitochondrial respiration, 2 μM oligomycin, 1 μM FCCP, and 0.5 μM each of rotenone and antimycin were added to each well.

### Bioinformatics.

We compared kidney tissue cell-specific gene expression using scRNA-Seq data available from the KPMP Kidney Tissue Atlas (30). At time of analysis, samples were available from living donors (LD, *n* = 20) and participants with diabetes (*n* = 11). The ‘FindMarkers’ function from the library ‘seurat’ was used for differentially expressed gene identification (47) using a restricted set of genes involved in the glycolysis, TCA cycle, mitochondrial respiration, and lactate transport pathway. No log fold change (logFC) threshold was set. Otherwise, default parameters were used. Comparisons were made using cell types as established by KPMP central analysis group (variable ‘subclass.l1’ in KPMP seurat object). ‘Subclass.l1’ includes multiple cell subtypes in some instances, named ‘subclass.l2’. Python version 3.11.2 (48) was used in conjunction with NumPy version 1.24.2 (49) and Pandas version 1.5.3 (50) to parse gene outputs and concatenate lists, respectively.

### Statistics.

Urine lactate was normalized to urine creatinine levels and log-transformed prior to analysis of the HUNT3 cohort. Between-patient comparisons were performed using the 2-tailed Welch’s *t* test at a 5% significance level. Correlation analyses between plasma lactate and plasma glucose in patients with DKD and healthy controls were performed using the Pearson’s correlation coefficient. A key endpoint studied in the SMART2D and CRIC cohorts was the change in eGFR over time (slope). Urine lactate levels were normalized to urine creatinine, log transformed, and grouped into tertiles for robustness against outliers and potentially nonlinear effects.

To model the change in eGFR, we fitted a linear mixed-effects model (random intercept and slope) with eGFR as the outcome and lactate tertiles alongside the covariates age, sex, ethnicity/race, BMI, HbA1c, mean arterial pressure (MAP), metformin use, baseline eGFR, and urine albumin/creatinine ratio (ACR, natural log-transformed) as predictors with a linear term for time. The estimate of interest was the interaction between time (year) and lactate tertile, and this represented the difference in eGFR slope associated with higher lactate levels. Next, for the purpose of causal inference, we performed a weighted, covariate balancing propensity score (CBPS) regression analysis (51) that compared participants in the lowest tertile of lactate with the upper 2 tertiles while equalizing other covariates. This type of causal inference estimates the counterfactual effect of patients moving from the lower to the upper lactate tertiles while keeping other covariates balanced. The baseline covariates balanced in this model were the same as the adjustment variables, except for time (age, sex, ethnicity/race, BMI, HbA1c, MAP, metformin use, baseline eGFR, and log-ACR). The R statistical software (Version 4.1, R Foundation for statistical computing) was used for both CRIC and SMART2D data sets.

To model the changes to urine and plasma lactate following acute exposure to hyperglycemia, we fitted a linear mixed-effects model for repeated measures under an unstructured random-effects covariance matrix for intercept and independence of errors. Each visit was considered a unit of repeated measure. Urine lactate and urine glucose were normalized to urine creatinine prior to analysis. Two-hypothesis testing with a significance level of 5% was used to assess the difference in changes to plasma and urine lactate levels following the euglycemic and hyperglycemic clamp. Specifically, the nonparametric Wilcoxon matched pairs signed rank test was performed. We also conducted correlation analyses using the Pearson’s correlation coefficient to assess the relationship between plasma and urine lactate with plasma glucose, as well urine lactate to urine glucose. To compare glucose uptake and lactate secretion in response to SGLT2 inhibition in isolated kidney sections, we performed a 1-way ANOVA test and corrected for multiple comparisons using the Tukey’s test. Oxygen consumption rate in kidney sections exposed to various lactate concentrations were analyzed using a 1-way ANOVA test and corrected for multiple comparisons using the Dunnett’s test. In all analyses conducted in this study, a *P* value less than 0.05 was considered significant. 

### Study approval.

Written informed consent was provided to all participants in SMART2D, HUNT3, CRIC, and ATIRMA cohorts prior to participation. The HUNT3 study was approved by the Regional Committee for Medical and Health Research Ethics of Norway. Ethics approval was given by the National Healthcare Group Domain Specific Review Board in Singapore for the SMART2D study. The CRIC study protocol was approved by IRBs at the participating institutions (refs. 18, 19) and was in accordance with the ethical principles of the Declaration of Helsinki. The ATIRMA study was approved by the University Health Network Research Ethics Board. Mouse study protocol reported in this paper have adhered to and have been approved by the IACUC.

### Data availability.

Data of participant biosamples in the CRIC study is available in NIDDK’s research repositories (https://repository.niddk.nih.gov/home/). Raw human data for SMART2D is available upon reasonable request from the Khoo Teck Puat Hospital data management office and ethical committee. All the measurement data and self-report data collected in HUNT3 is stored in HUNT Databank (https://www.ntnu.edu/hunt/databank). The KPMP database is freely accessible and publicly available to use at https://www.kpmp.org All raw data values for graphs are provided in the Supporting Data Values file. Any additional information, including supporting analytical code, is available from the corresponding authors upon reasonable request.

## Author contributions

KS, JG, and DZIC designed the study. MD, LK, SM, VSS, RF, AS, JJL, and SH analyzed the data. MD, LK, SM, VSS, and JW made the figures. MD, LK, SM, VSS, RF, CPL, BIG, AS, GZ, VRD, JJK, RM, JT, DM, ZW, JJL, JW, BAP, YL, LN, SCL, HF, RT, JRS, JP, SSW, JB, YO, JH, JC, WBR, IHdB, SR, VV, SH, JALG, DZIC, and KS have contributed to the writing of the manuscript and provided critical edits to, reviewed, and approved the final manuscript. KS is the guarantor of this work.

## Supplementary Material

Supplemental data

Supporting data values

## Figures and Tables

**Table 1:**
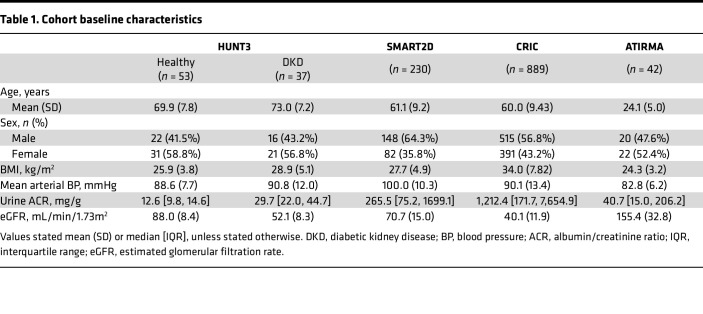
Cohort baseline characteristics

**Table 2:**
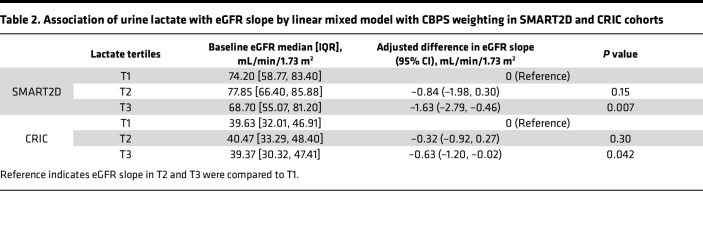
Association of urine lactate with eGFR slope by linear mixed model with CBPS weighting in SMART2D and CRIC cohorts

**Table 3:**
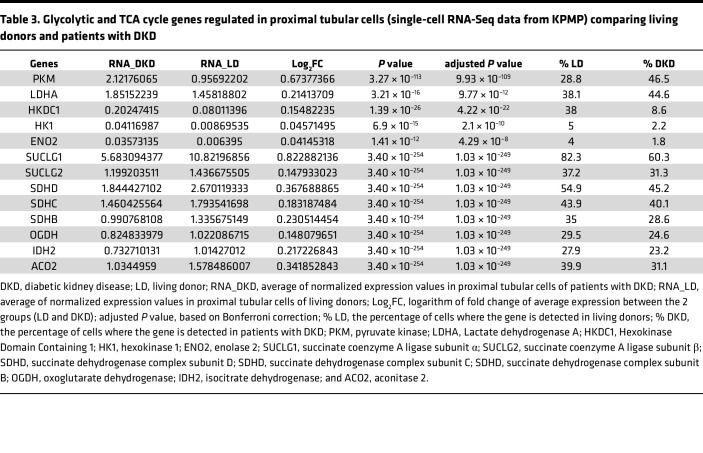
Glycolytic and TCA cycle genes regulated in proximal tubular cells (single-cell RNA-Seq data from KPMP) comparing living donors and patients with DKD

## Version 1 06/10/2024

Electronic publication
