# Supplementary material for: Glycolytic lactate in diabetic kidney disease
Source: JCI Insight. 2024 Jun 10;9(11):e168825. doi: 10.1172/jci.insight.168825 (PMC11382878; doi:10.1172/jci.insight.168825)
Supplement: Supplemental data [file jciinsight-9-168825-s097.pdf]

Supplemental Acknowledgments

CRIC study investigators:

Lawrence J. Appel[1], Alan S. Go[2,3], James P Lash[4], Robert G. Nelson[5], Mahboob Rahman[6], Panduranga A. Rao[7], Vallanbh O Shah[8], Debbie L Cohen[9], and Mark L. Unruh[10]

[1]Department of Epidemiology and Welch Center for Prevention, Epidemiology, and Clinical Research, Bloomberg School of Public Health, Johns Hopkins University, Baltimore, Maryland.
[2]Division of Nephrology, UCSF School of Medicine, San Francisco, California, USA.
[3]Division of Research, Kaiser Permanente Northern California, Oakland, California, USA.
[4]Department of Medicine, University of Illinois, Chicago, Illinois.
[5]Chronic Kidney Disease Section, Phoenix Epidemiology and Clinical Research Branch, National Institute of Diabetes and Digestive and Kidney Diseases, National Institutes of Health, Phoenix, Arizona
[6]Division of Nephrology and Hypertension, University Hospital Cleveland Medical Center, Cleveland, Ohio.
[7]Department of Medicine, University of Michigan Health System, Ann Arbor, Michigan, USA.
[8]Department of Biochemistry and Molecular Biology, School of Medicine, University of New Mexico Health Sciences Center, Albuquerque, New Mexico, USA.
[9]Department of Medicine, Perelman School of Medicine, University of Pennsylvania, Philadelphia.
[10]Department of Internal Medicine, School of Medicine, University of New Mexico, Albuquerque, USA.

Kidney Precision Medicine Project:

Richard Knight, Stewart Lecker, Isaac Stillman, Sushrut Waikar, Gearoid Mcmahon, Astrid Weins, Samuel Short, Nir Hacohen, Paul Hoover, Mark Aulisio, Leslie Cooperman, Leal Herlitz, John O'Toole, Emilio Poggio, John Sedor, Stacey Jolly, Paul Appelbaum, Olivia Balderes, Jonathan Barasch, Andrew Bomback, Pietro A Canetta, Vivette D d'Agati, Krzysztof Kiryluk, Satoru Kudose, Karla Mehl, Jai Radhakrishnan, Chenhua Weng, Laura Barisoni, Theodore Alexandrov, Tarek Ashkar, Daria Barwinska, Pierre Dagher, Kenneth Dunn, Michael Eadon, Michael Ferkowicz, Katherine Kelly, Timothy Sutton, Seth Winfree, Steven Menez, Chirag Parikh, Avi Rosenberg, Pam Villalobos, Rubab Malik, Derek Fine, Mohammed Atta, Jose Manuel Monroy Trujillo, Alison Slack, Sylvia Rosas, Mark Williams, Evren Azeloglu, Cijang John He, Ravi Iyengar, Jens Hansen, Samir Parikh, Brad Rovin, Chris Anderton, Ljiljana Pasa-Tolic, Dusan Velickovic, Jessica Lukowski, George Holt Oliver, Joseph Ardayfio, Jack Bebiak, Keith Brown, Taneisha Campbell, Catherine Campbell, Lynda Hayashi, Nichole Jefferson, Robert Koewler, Glenda Roberts, John Saul, Anna Shpigel, Edith Christine Stutzke, Lorenda Wright, Leslie Miegs, Roy Pinkeney, Rachel Sealfon, Olga Troyanskaya, Katherine Tuttle,

Dejan Dobi, Yury Goltsev, Blue Lake, Kun Zhang, Maria Joanes, Zoltan Laszik, Andrew Schroeder, Minnie Sarwal, Tara Sigdel, Ulysses Balis, Victoria Blanc, Oliver He, Jeffrey Hodgin, Matthias Kretzler, Laura Mariani, Rajasree Menon, Edgar Otto, Jennifer Schaub, Becky Steck, Chrysta Lienczewski, Sean Eddy, Michele Elder, Daniel Hall, John Kellum, Mary Kruth, Raghav Murugan, Paul Palevsky, Parmjeet Randhawa, Matthew Rosengart, Sunny Sims-Lucas, Mary Stefanick, Stacy Stull, Mitchell Tublin, Charles Alpers, Ian de Boer, Ashveena Dighe, Jonathan Himmelfarb, Robyn Mcclelland, Sean Mooney, Stuart Shankland, Kayleen Williams, Kristina Blank, Jonas Carson, Frederick Dowd, Zach Drager, Christopher Park, Kumar Sharma, Guanshi Zhang, Shweta Bansal, Manjeri Venkatachalam, Asra Kermani, Simon Lee, Christopher Lu, Tyler Miller, Orson Moe, Harold Park, Kamalanathan Sambandam, Francisco Sanchez, Jose Torrealba, Toto Robert, Miguel Vazquez, Nancy Wang, Joe Gaut, Sanjay Jain, Anitha Vijayan, Randy Luciano, Dennis Moledina, Ugwuowo Ugochukwu, Francis Perry Wilson, Sandy Alfano

Supplemental Table 1: Demographic and baseline characteristics of living donors and patients with DKD from KPMP data.

	LD (n=20)	DKD (n=11)
**Age, n (%)**		
30-39 years	6 (30%)	2 (18.2%)
40-49 years	6 (30%)	0
50-59 years	7 (35%)	1 (9.1%)
60-69 years	1 (5%)	4 (36.4%)
70-70 years	0	4 (36.4%)
**Sex, n (%)**		
Male	7 (35%)	3 (27.3%)
Female	13 (65%)	8 (72.7%)
**Diabetes Duration, n (%)**		
0-4 years	-	1 (9.1%)
5-9 years	-	1 (9.1%)
10-14 years	-	3 (27.3%)
20-24 years	-	4 (36.4%)
25-29 years	-	1 (9.1%)
30-34 years	-	1 (9.1%)
**HbA1c, n (%)**		
<6.5%	-	1 (9.1%)
6.5% to <7.5%	-	5 (45.5%)
7.5% to <8.5%	-	0
<8.5%	-	2 (18.2%)
Unavailable data	20 (100%)	3 (27.3%)
**eGFR, n (%)**	Not available	
20-60 ml/min/1.73m^2	-	8 (72%)
>60 ml/min/1.73m^2	-	3 (28%)
**UACR, n (%)**		
<30 mg/g	-	2 (18.2%)
30 to <300 mg/g	-	1 (9.1%)
500 to <1000 mg/g	-	1 (9.1%)
>=1000 mg/g	-	2 (18.2%)
Unavailable data	20 (100%)	5 (45.5%)
**Use of RAAS blockade, n (%)**	Not available	
**Yes**	-	6 (54.5%)
**No**	-	5 (45.5%)
**History of hypertension, n (%)**	Not available	

**Yes**	-	10 (90.9%)
**No**	-	1 (9.1%)

DKD, diabetic kidney disease; LD living donor; HbA1c, hemoglobin A1c; eGFR, estimated glomerular filtration rate; UACR, urine albumin-to-creatinine ratio; RAAS, renin-angiotensin-aldosterone system.

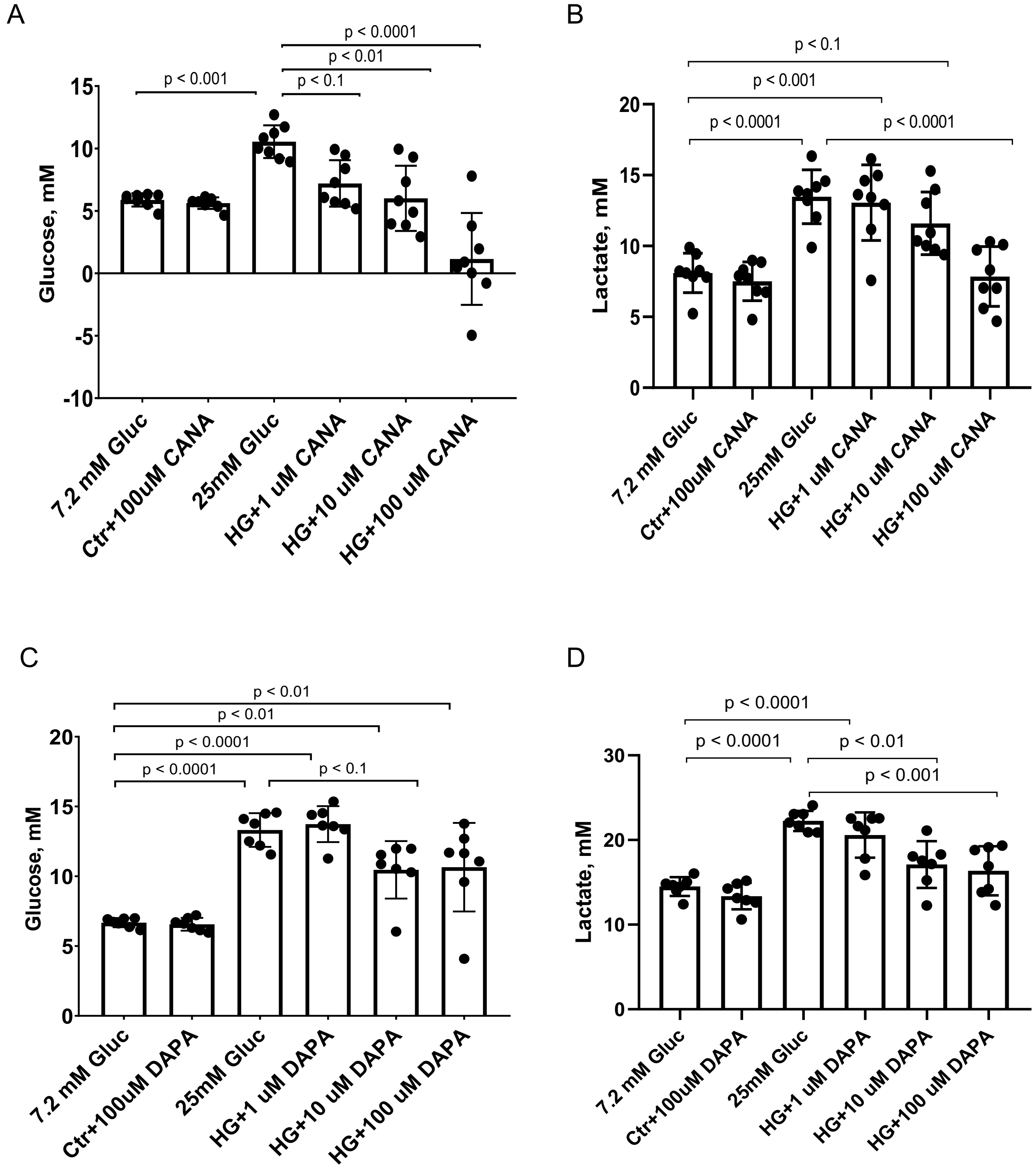

Supplemental Figure 1: Glucose induced increase in lactate in kidney sections is reduced by canagliflozin and dapagliflozin. Mouse kidney sections from 10–12-week-old male C57Blk/6J mice have a SGLT2 dependent increase in glucose (Gluc) uptake from normal glucose (NG) to high glucose (HG) (panel A, C) and lactate production (panel B, D). The SGLT2 inhibitor, Canagliflozin (CANA) and Dapagliflozin (DAPA) exhibit a dose dependent effect to reduce glucose uptake and lactate secretion compared to control (CTR).

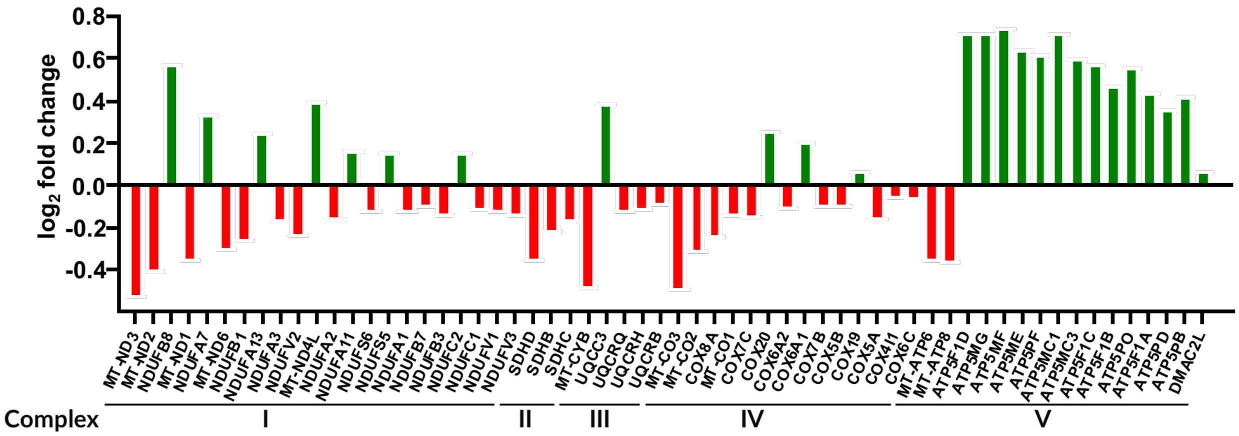

Supplemental Figure 2. **Expression of respiratory genes in the proximal tubular (PT) cells of patients with diabetic kidney disease (DKD).** Log2 fold-change calculated between average of normalized gene expression values form the living donors (LD; n=20) and DKD patients (n=11) in PT cells. Compared to LD, positive log2 fold-change indicate upregulated and negative values indicate downregulated genes in DKD.

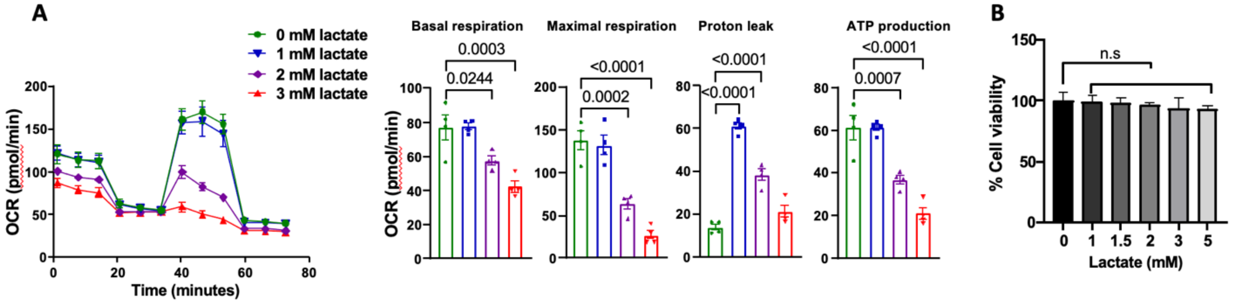

Supplemental Figure 3. Extracellular lactate inhibits OCR in isolated mouse proximal tubular cells in a dose-dependent manner without cytotoxicity. OCR was measured in mouse primary renal tubular epithelial cells using Seahorse extracellular flux analyzer with 1-hour preincubation with different concentration (0-3mM). Basal respiration, maximal respiration, proton leak and ATP production linked OCR were calculated from the above traces (panel A). MTT assay in HK2 cells treated with various concentration of lactate for 24h (n=4) (panel B).

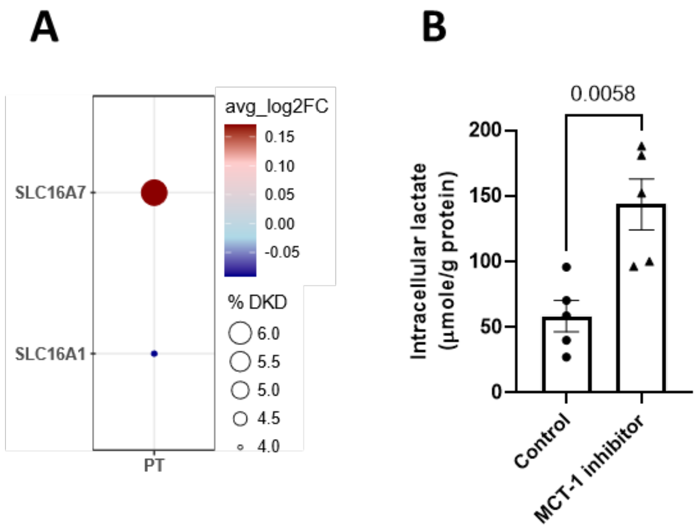

Supplemental Figure 4. Inhibition of lactate transporter accumulates intracellular lactate in kidney cortical sections. Dot plot of regulated lactate transporters, MCT-1(SLC16A1) and MCT2 (SLC16-A7) in the proximal tubular (PT) cells of patients with diabetic kidney disease (DKD). Log2 fold-change calculated between the average of normalized gene expression values from the living donors (LD; n=20) and DKD patients (n=11) in PT cells. % DKD circle size shows the percentage of cells in which the gene was detected within in DKD biopsies (panel A). Kidney sections from 10–12-week-old male C57Blk/6 mice were treated with 2 µm of MCT-1 inhibitor (SR13800, Sigma) for 24h and measured the cellular lactate level (panel B).

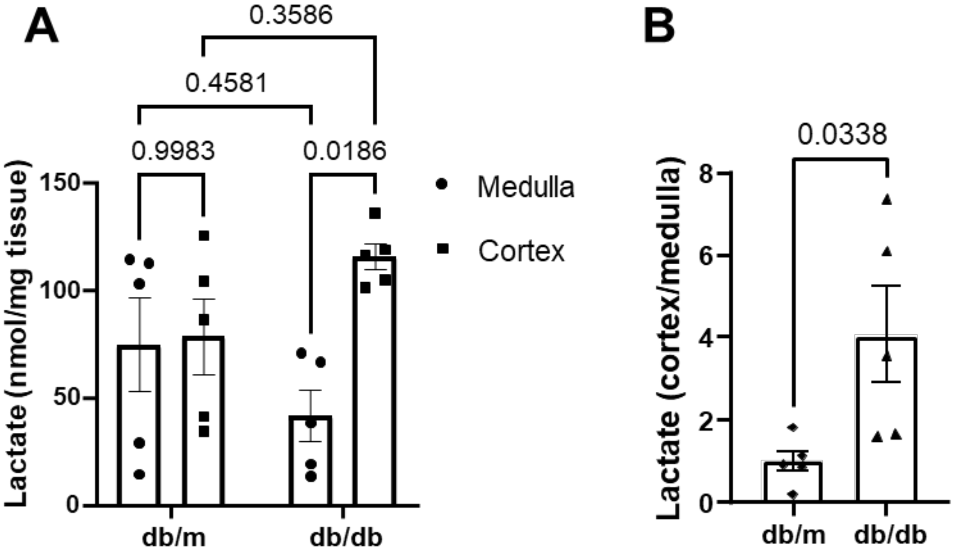

Supplemental Figure 5. Kidney lactate level in diabetic mice. Lactate levels and cortex/medulla lactate ratio in kidney medulla and cortex were compared between 6-month-old db/m and db/db mice (n=5/group). Two-way ANOVA followed by Tukey's multiple comparison test was performed for statistical analysis (panel A). Student 't' test was performed for statistical analysis (panel B).
